# Disseminating information systems across the Atlantic: Collaboration between U.K. National Health Service and U.S. Department of Veterans Affairs

**DOI:** 10.1186/1748-5908-10-S1-A62

**Published:** 2015-08-20

**Authors:** D Keith McInnes, Thomas K Houston, Susan S Woods, Kathleen L Frisbee, Neil C Evans

**Affiliations:** 1 Center for Healthcare Organization and Implementation Research, VA New England Healthcare System, Bedford, MA 01730, USA; 2 eHealth Quality Enhancement Research Initiative, VA New England Healthcare System, Bedford, MA 01730, USA; 3 Maine VA Healthcare System, Portland, Maine, 04101, USA; 4 Connected Health Office, Veterans Health Administration, Washington, D.C., 20420, USA

## Introduction

Patients with chronic health conditions benefit from frequent monitoring and encouragement to maintain good health. Virtual care technologies (mHealth, texting) tethered to electronic health records provide a platform for frequent communication. After implementing "Florence," referred to as Flo, a text messaging system to enable patients to report measures such as blood pressure, the United Kingdom's National Health Service (NHS) worked with the US Department of Veterans Affairs (VA) to develop a similar system, named "Annie" after Annie Fox, a military nurse and first woman to receive the Purple Heart.

## Methods

NHS and VA consulted via email, conference calls, and an in-person seminar to inform design and implementation in VA. At the seminar NHS presented 10 case studies of Florence, and break-out groups listed lessons learned. Also informing the design of Annie, pre-implementation studies were conducted including a survey and live text-messaging with Veterans to assess acceptability, usability, and usefulness.

## Results

Lessons learned from NHS included that the system should be customizable by clinicians and administrators, fit patients' daily lives, and have a simple user interface. In pre-implementation studies, participants (n = 106) reported they own cell phones (89%), of which 5% are smart phones. Most respondents (88%) were interested in text messaging for health purposes. In a test of text message appointment reminders, usability and ease of use were highly rated, and over 90% of Veterans wanted the service to continue. Downward trends were observed for patient missed appointments, cancelled appointments, and emergency room use.

## Discussion

Highly collaborative, cross-national dissemination improved and accelerated VA implementation of a patient communication system. Empiric and pilot data (from both countries) informed the development and garnered leadership support for the system. Lessons from VA's implementation will flow back to Florence, in a collaborative process improvement cycle.

## Primary source of funding

US Department of Veterans Affairs

